# Advances in Genetic Diagnosis of Kallmann Syndrome and Genetic Interruption

**DOI:** 10.1007/s43032-021-00638-8

**Published:** 2021-07-06

**Authors:** Yujun Liu, Xu Zhi

**Affiliations:** 1 Center for Reproductive Medicine, Department of Obstetrics and Gynecology, Peking University Third Hospital, Beijing, 100191 China; 2 National Clinical Research Center for Obstetrics and Gynecology, Peking University Third Hospital, Beijing, 100191 China; 3 Key Laboratory of Assisted Reproduction (Peking University, Ministry of Education, Beijing, 100191 China; 4 Beijing Key Laboratory of Reproductive Endocrinology and Assisted Reproductive Technology, Beijing, 100191 China

**Keywords:** Kallmann syndrome, Genetic diagnosis, Prenatal diagnosis, Preimplantation genetic testing

## Abstract

Kallmann syndrome (KS) is a rare hereditary disease with high phenotypic and genetic heterogeneity. Congenital hypogonadotropic hypogonadism and hyposmia/anosmia are the two major characterized phenotypes of KS. Besides, mirror movements, dental agenesis, digital bone abnormalities, unilateral renal agenesis, midline facial defects, hearing loss, and eye movement abnormalities can also be observed in KS patients. Because of the phenotypic heterogeneity, genetic diagnosis become increasingly valuable to distinguish KS from other disorders including normosmic congenital hypogonadotropic hypogonadism, constitutional delay of growth and puberty, CHARGE syndrome, and functional hypogonadotropic hypogonadism. Application of next-generation sequencing has promoted the discovery of novel pathogenic genes in KS pedigrees. Prenatal diagnosis is an effective method in clinical settings to decrease birth defects and block transmission of genetic disorders. However, pregnant women may suffer from physical and psychological distress when fetuses are diagnosed with congenital defects. Preimplantation genetic testing (PGT) is a prospective approach during the in vitro fertilization process that helps to interrupt transmission of hereditary diseases to offspring at an early stage. Thus, genetic testing and counseling are recommended to KS patients with family histories, prenatal diagnosis and PGT are considered to be useful options.

## Introduction

Kallmann syndrome (KS) is a hereditary disease characterized by congenital hypogonadotropic hypogonadism (CHH) and hyposmia or anosmia. Isolated gonadotropin-releasing hormone (GnRH) deficiency (IGD) is a common disease with genetic and clinical heterogeneity, and KS is the main component of IGD. The estimated incidence of KS in males (1:30,000) is 4–5 times higher than in females (1:125,000), but the latter is probably underestimated because affected women tend to have milder symptoms than affected men [1]. KS can be sporadic or familial, and approximately 60% of cases present in a sporadic pattern. KS patients exhibit high phenotypic and genotypic heterogeneity, and thus genetic testing provides crucial evidence for diagnosis and guidance of treatment. Approximately 40% of KS patients have one or several rare sequence variants that have been identified [2], while the remaining cases have no identified genetic cause. KS was traditionally considered as a monogenic disorder, but in recent years, the monogenic paradigm was challenged with the digenic and oligogenic modes following the advanced understanding of KS, indicating that KS is not strictly a monogenic Mendelian disease. Therefore, although most pathogenic genes identified in KS follow autosomal dominant, autosomal recessive, or X-linked inheritance patterns, a small number of the genes also inherited in digenic and oligogenic modes [3]. Infertility is one of the problems affecting normal life in male KS patients, and the majority of male patients tend to seek for help for reproductive treatments. Owing to the hereditary nature of KS, genetic counseling, and hereditary risk evaluation are recommended. Prenatal screening/diagnosis is an effective method in clinical settings to decrease birth defects and block transmission of genetic disorders. However, pregnant women suffer from physical and psychological distress when fetuses are diagnosed with KS, and they experience induced abortion. With the deepening of related research, advances have been made in explorations of molecular genetics in KS, especially after the invention of next generation sequencing (NGS). The emergence of preimplantation genetic testing (PGT) has provided families affected by hereditary disorders (including KS) with an optional approach to have healthy offspring. This review summarizes the advances in KS, especially its genetic diagnosis, and genetic interruption of KS in clinical settings.

## General Description of KS and Advances in Genetic Diagnosis

### Discovery and Causes of KS

CHH and hyposmia or anosmia are the two typical symptoms of KS. The correlation between anosmia and small testes in individuals was first noted by a Spanish pathologist, Aureliano Maestre de San Juan, in 1856 [4]. In 1944, American geneticist Franz Jozef Kallmann first described KS in three families and suggested a hereditary background [5].

GnRH or luteinizing hormone-releasing hormone (LHRH), a decapeptide, is essential for mammalian puberty onset and reproduction. The reproductive function is mainly managed by 1200–1500 GnRH neurons, and these unique neurons have either an olfactory placode/ectodermal or a neural crest cell origin [6–8]. GnRH is produced by the arcuate nucleus in the hypothalamus and delivered to the anterior pituitary through pituitary portal blood vessels, where it pulsatively stimulates the synthesis and secretion of luteinizing hormone (LH) and follicle-stimulating hormone (FSH) that participate in gonadal maturation and function in both men and women [9, 10].

In 1989, Schwanzel-Fukuda et al. [11] and Wray et al. [12] proved that GnRH neurons were unusual neuroendocrine cells derived from progenitor cells outside the central nervous system in the medial olfactory placode that migrated across the nasal septum, entered the forebrain with the nervus terminalis, and finally arched into the septal-preoptic area and hypothalamus. Several years later, similar spatiotemporal migration and development patterns of GnRH neurons were confirmed in early human embryos [13, 14]. KS, a neuronal migration defect in humans, is caused by disruption of olfactory axon development and GnRH neuron migration. LHRH-expressing cells were found to be absent in the brain of KS fetuses, although dense clusters of LHRH cells and fibers were present in the nose [15]. The developmental relationship between the GnRH system and the olfactory system illustrates some of the pathogenesis and clinical symptoms in KS patients.

The hypothalamic-pituitary-gonadal (HPG) axis is activated from the mid-trimester, restrained at the end of gestation, and reactivated after birth. In early childhood (6 months of age in boys; 3–4 years of age in girls), GnRH pulsatile secretion is actively inhibited and persists until adolescence [16]. Reactivation of GnRH release after this quiescent period marks the initiation of puberty [16], but the mechanisms involved in triggering of puberty remain unclear. During puberty, GnRH regulates the synthesis and release of FSH and LH. In boys, FSH promotes proliferation of immature Sertoli cells and spermatogonia, while LH stimulates Leydig cells to produce testosterone, leading to initiation of spermatogenesis [17]. In girls, FSH and LH are crucial for follicular maturation and ovulation.

### Frequent and Rare Clinical Symptoms of KS

CHH and hyposmia or anosmia are the most typical characteristics of KS. Because HPG axis activity and function fluctuate during the lifespan, the axis provides a good time-window for diagnosis of KS. Based on the influence of GnRH participation in different periods of growth and development in humans, the symptoms of KS can be summarized into three stages: (1) neonatal period and childhood, (2) adolescence, and (3) adulthood. *Neonatal period and childhood* In male infants, cryptorchidism and micropenis (stretched penile length <2.5 cm) [18, 19] are the common signs of GnRH deficiency during the neonatal period and childhood [20]. In GnRH-deficient female neonates, no specific clinical signs are present at this stage. *Adolescence* Because of their low GnRH levels, KS patients show absent or partial pubertal development in adolescence. In male adolescents, absent and/or minimal virilization, low libido, and lack of sexual function are the common symptoms [20]. Absence of breast development and/or primary amenorrhea are the most common complaints in female adolescents [20]. *Adulthood* KS diagnosis in adulthood is mainly associated with infertility and sometimes with early appearance of osteoporotic fractures [20, 21]. Some male KS patients suffer from increased breasts or no beard, which cause psychological problems and affect their normal life. For female KS adults, besides the two characteristic symptoms (CHH and hyposmia/anosmia), congenital heart disease (including fatigue, dyspnea, cyanosis, palpitations, syncope) and neurologic manifestation (including hearing defect, epilepsy, paraplegia) may be found in a minority of females with KS, but the symptoms in females are milder than males.

Besides the symptoms mentioned above, small numbers of KS patients show synkinesia (mirror movements), dental agenesis, digital bone abnormalities (polydactyly, syndactyly, campylodactyly), unilateral renal agenesis or urinary tract malformation, midline facial defects (cleft lip/palate), hearing loss, eye movement abnormalities, poor balance through cerebellar ataxia, skeletal anomalies (scoliosis, polydactyly, clinodactyly), and pigmentation defects [22–25].

The psychological problems of KS patients are non-ignorable, especially among male adolescents, and include depression, anxiety, and decreased quality of life that leads to low self-esteem and other effects [26]. Compared with control patients, male CHH patients were reported to have significantly high scores in a series of test scales, including the Beck Depression Inventory (BDI), Beck Anxiety Inventory, and Arizona Sexual Experiences (ASEX), indicating noteworthy psychological problems [26]. After 6 months of hormone replacement therapy (HRT), the BDI and ASEX scores and psychological symptoms were improved [26]. Because of the self-contempt, KS patients may hesitate to communicate with others, particularly in adolescents who are suffering from emotional fluctuation. Thus, besides medical treatments for painful physical symptoms, psychological care is an indispensable component of the treatment strategy for KS patients.

### Diagnosis of KS

In the first several months after birth, transient activation of the HPG axis is defined as “mini-puberty” [27]. During mini-puberty, testosterone is increased in both boys and girls, and girls also have increased estradiol. Such mini-puberty is observed at 1–3 months of age for the diagnosis of IGD. Besides mini-puberty, IGD is mostly diagnosed during adolescence or early adulthood.

The clinical diagnosis of KS includes evaluation of CHH and hyposmia or anosmia. The diagnosis of KS can rely on reproductive symptoms, such as history of cryptorchidism and/or micropenis [28], presence of arrested sexual maturation and/or lack of secondary sexual characteristics, diminished libido, and infertility [25]. Because of the unreliability of self-reporting of a normal sense of smell, formal smell tests are necessary for evaluation of hyposmia or anosmia [29, 30]. Besides the two most common symptoms, some KS patients have other organ complaints such as bimanual synkinesia, dental agenesis, digital bone abnormalities, unilateral renal agenesis or urinary tract malformation, hearing loss, skeletal anomalies, pigmentation defects, and eye movement abnormalities [25, 28].

Hormonal analysis provides useful indicators for KS diagnosis. Male and female KS patients both have very low plasma gonadotrophin, FSH, LH, and inhibin B levels. During mini-puberty in particular, serum levels of testosterone (male infants) and FSH and LH (male and female infants) show transient increases that are extremely low in infants with CHH [31].

Meanwhile, some brain changes have been noted in KS patients. Magnetic resonance imaging examination can show abnormalities in the olfactory fossae, such as unilateral or bilateral olfactory bulb or tract agenesis, reduced depth, reduced curvature, or increased cortical thickness within the olfactory sulcus in KS patients [30, 32, 33]. However, a minority of KS patients with confirmed anosmia show the normal olfactory structure. The anosmia or hyposmia in these patients may be caused by other reasons such as viral infections, trauma, or drug-induced smell disturbance.

### Genetic Diagnosis of KS

Although the majority of KS cases are sporadic, many cases are clearly familial, and more than 30 mutated genes in KS patients have been identified to date [20]. These genes either act alone (autosomal dominant [AD], autosomal recessive [AR], and X-linked inheritance patterns) or in combination (digenic or oligogenic modes) [3]. The mutated genes are summarized below.

*ANOS1* (also known as *KAL1*), the first and most common pathogenic gene for KS identified in 1991, is inherited through the X-linked recessive mode [34]. *ANOS1*, located on Xp22.3 and consisting of 14 exons, encodes the protein anosmin-1, a transient and regionally restricted extracellular adhesion protein that participates in GnRH neuron adhesion and axonal migration during organogenesis [34, 35]. Anosmin-1 is expressed from the early stage of pregnancy in the region of the olfactory bulb [36]. Nucleotide insertions and deletions are the main mutation types detected in *ANOS1* and cause frameshifts or premature termination codons during protein translation process [37]. A 19-week human male fetus with a deletion in *ANOS1* showed disconnection of olfactory, vomeronasal, and terminalis nerve fibers with the brain, and GnRH neurons were unable to migrate to their normal position in the brain [15, 36]. *ANOS1* mutations are found in 10%–20% of all KS patients, including 30%–60% of familial cases and 10%–15% of sporadic cases [38–40].

*FGFR1* was reported to be mutated in KS families by Dodé et al. in 2003, and *FGFR1* mutations are the second most common gene mutations found in KS patients. *FGFR1* encodes fibroblast growth factor receptor 1 (FGFR1), which belongs to the receptor tyrosine kinase superfamily [41, 42]. The incidence of *FGFR1* mutations in KS patients is ~10% [37]. *FGFR1* is located on chromosome 8p11.23 and follows the AD/AR/oligogenic inheritance modes. FGFR1 consists of an extracellular domain (three immunoglobulin-like domains that determine the affinity and specificity for its ligands), a transmembrane helix domain, and an intracellular tyrosine kinase domain [43]. *FGFR1* mutations include missense, nonsense, splice site, frameshift mutations and intragenic insertions or deletions, and the phenotypes arising from these different mutations are heterogeneous [43–45]. *FGFR1* mutations are associated with delayed puberty, anosmia, cleft palate, mirror movements, dental agenesis, and bimanual synkinesia, although some familial cases have males and females with FGFR1 mutations who are asymptomatic carriers [42, 46–48]. There is no experimental evidence for a potential dominant-negative effect of mutated receptors on wild-type FGFR1, and thus it is speculated that the phenotypes mainly result from haploinsufficiency [43]. Surprisingly, a KS patient with an *FGFR1* mutation was reported to show a reversible condition after several years of testosterone treatment, and the same mutation was found in his mother and maternal grandfather [45]. However, the mechanisms behind the *FGFR1* mutation dominant and recessive forms in KS remain unclear.

*PROKR2* and *PROK2* encode the G-protein-coupled receptor prokineticin receptor 2 (PROKR2) and its ligand prokineticin-2 (PROK2), respectively, and the PROKR2/PROK2 pathway plays essential roles during olfactory bulb development and GnRH neuronal progenitor migration and differentiation [49]. *PROKR2* and *PROK2* mutations can be heterozygous, homozygous, or compound heterozygous, and the prevalence of these mutations in KS patients is about 5%–10% [37, 50]. *Prokr2* or *Prok2* knockout mice were found to show hypoplasia of the olfactory bulb and reproductive system, without the normal architecture [51, 52]. KS patients who carry loss-of-function mutations in these two genes show different degrees of CHH and hyposmia or anosmia, coincident with the findings in mice [50, 52, 53].

*CHD7* located on chromosome 8q12 consists of 38 exons, and encodes chromodomain helicase DNA-binding protein 7 (CHD7). Mutations of *CHD7* were identified in patients with CHARGE syndrome, KS, and CHH [54]. Although *CHD7* was originally considered to be the causative gene for CHARGE syndrome, subsequent research showed that *CHD7* mutations can also be detected in KS patients lacking mutations in *KAL1*, *FGFR1*, *PROK2*, and *PROKR2* [55]. The prevalence of *CHD7* mutations in all KS/nIHH patients is predicted to be >5% [56, 57].

As well as the major genes described above, the development of NGS has led to the identification of many other pathogenic genes in KS patients during the last several decades. The majority of the mutated genes identified to date participate in GnRH neuron development and migration or secretion processes. *FGF8/FGFR1*, *SOX2*, *CHD7*, *FGF17*, and *IL17RD* are involved in the GnRH neuron fate specification process [20], while *ANOS1*, *PROK2/PROKR2*, *SEMA3A/PLXNA1*, *SEMA3E*, *NSMF (NELF)*, *HS6ST1*, *WDR11*, *SOX10*, *FEZF1*, *IGSF10*, *DCC/NTN1*, *TUBB3*, and *SMCHD1* are associated with olfactory axon guidance, GnRH neuron migration, or axon projection [46] (Table 1).

**Table 1:** Pathogenic genes identified in KS

Genes	OMIM	Description	Chromosome	Inheritance pattern	Functions	Clinical phenotypes	Mutation in other disorders
*ANOS1 (KAL-1)*	300836	Anosmin-1	Xp22.31	XLR	Development and migration of olfactory and GnRH neurons	Delayed puberty, anosmia, micropenis, hearing loss, mirror movement, unilateral renal agenesis/hypoplasia	-
*FGFR1* *(KAL-2)*	147950	Fibroblast growth factor receptor 1	Chr8p11.23	AR/AD/Oligo	Development and migration of olfactory and GnRH neurons	Delayed puberty, anosmia, bimanual synkinesia, cleft palate, mirror movements, and dental agenesis	nCHH, CPHD, split-hand/foot malformation, Hartsfield syndrome, Pfeiffer syndrome, Jackson-Weiss syndrome, Antley-Bixler syndrome, osteoglophonic dysplasia, craniosynostosis
*PROK2*	610628	Prokineticin 2	Chr3p21.1	AR/AD/Oligo	GnRH neuron migration	Obesity, epilepsy, sleep disorders, fibrous dysplasia, and synkinesia	nCHH
*PROKR2*	244200	Prokineticin receptor 2	Chr20p12.3	AR/AD/Oligo	Olfactory and GnRH neuron migration	Obesity, sleep disorders, pectus excavatum, pes planus, synkinesia, seizures, hyperlaxity of digits	nCHH, CPHD, Morning Glory syndrome
*IL17RD*	615267	Interleukin 17 receptor D	Chr3p14.3	AR/AD/DD	GnRH neuron fate specification	Hearing loss, osteopenia, osteoporosis	-
*CHD7*	612370	Chromodomain-helicase-DNA-binding protein 7	Chr8q12.2	AD	GnRH neuron fate specification	Sensorineural deafness, hyposmia or anosmia, cleft lip/palate, cryptorchidism, hypoestrogenic amenorrhea	CHARGE syndrome, nCHH
*FGF8*	612702	Fibroblast growth factor 8	Chr10q24.32	AD/Oligo	GnRH neuron fate specification	High-arched palate, cleft lip/palate, mid-line defects, micropenis	nCHH, CPHD
*FGF17*	603725	Fibroblast growth factor 17	Chr8p21.3	AD/Oligo	GnRH neuron migration	-	Dandy-Walker syndrome
*WDR11*	614858	WD repeat domain 11	Chr10q26.12	AD	Development of GnRH neurons	Obesity, ciliopathy,	nCHH, CPHD
*SEMA3A*	614897	Semaphorin 3A	Chr7q21.11	AD/Oligo	Axonal path finding of GnRH neurons	Anosmia, delayed/absent puberty, knee valgus, transverse palm	nCHH, CHARGE
*SEMA3E*	608166	Semaphorin 3E	Chr7q21.11	AD/Oligo	GnRH neuron migration	Anosmia, cleft lip/palate, microcephaly, facial asymmetry, hearing loss, micropenis, colobomas	nCHH, CHARGE
*SEMA7A*	607961	Semaphorin 7A	Chr15q24.1	-	GnRH neuron migration	Primary amenorrhea, hyposmia/anosmia	nCHH
*SOX10*	602229	SRY-box transcription factor 10	Chr22q13.1	AD	GnRH neurons migration	Hyposmia/anosmia, hearing loss	Waardenburg syndrome
*HS6ST1*	614880	Heparan sulfate 6-O-sulfotransferase 1	Chr2q14.3	AD/Oligo	GnRH neuron migration	Anosmia, cleft palate, micropenis, small testes, cryptorchidism, genu valgus, osteopenia or osteoporosis	nCHH
*NSMF (NELF)*	614838	NMDA receptor synaptonuclear signaling and neuronal migration factor	Chr9q34.3	AD/Oligo	GnRH neuron migration	Hyposmia/anosmia, cleft/palate, clinodactyly, osteoporosis	nCHH, CPHD
*FEZF1*	613301	FEZ family zinc finger 1	Chr7q31.32	AR	GnRH neuron migration	Anosmia, delayed/absent puberty, micropenis	-
*PLXNA1*	601055	Plexin A1	Chr3q21.3	AR/Oligo	GnRH neuron migration	Delayed puberty, anosmia	nCHH
*SPRY4*	607984	Sprouty homolog interactor with FGFR1	Chr5q31.3	AD/Oligo	GnRH neuron migration	Delayed/ absent puberty, anosmia/hyposmia, hearing loss, abnormal dentition,	nCHH
*FLRT3*	604808	Fibronectin like domain containing leucine enrich transmembrane protein 3	Chr20p12.1	AD	GnRH neuron migration	Anosmia, delayed puberty, cleft palate	nCHH
*DUSP6*	602748	Dual specific inhibitor phosphatases	Chr12q21.33	AR	GnRH neuron migration	Cryptorchidism, hearing loss, dental agenesis, syndactyly and blue color blindness	nCHH
*DCC*	120470	DCC Netrin 1 receptor	Chr18q21.2	AR/AD/Oligo	GnRH migration/ axon guidance	Medline defects, scoliosis, mirror movement, hyperreflexia, and other CNS symptoms	nCHH
*NTN1*	601614	*Netrin 1*	Chr17p13.1	AD/Oligo	GnRH migration/ axon guidance	Medline defects, mirror movement	nCHH
*HESX1*	182230	Homeobox gene expressed in ES cells	Chr3p14.3	AD/AR	GnRH neural development, migration, and function	Short stature, optic nerve hypoplasia, hypoplastic optic discs, supernumerary digits, hypoplastic digits, mid-line defects	CPHD, septo-optic dysplasia
*AXL*	109135	AXL receptor tyrosine kinase	Chr19q13.2	-	GnRH neuron migration	Anosmia, delayed puberty, cleft lip/palate	nCHH

*AD* accessive dominant, *AR* accessive recessive, *Oligo* oligogenic

In addition to the classical Mendelian inheritance pattern, digenic and oligogenic modes have been described in KS patients [58]. First mentioned in 1994, digenic or oligogenic transmission refers to two or more than two synergic deleterious gene mutations in an individual that contribute to a disease phenotype [59]. Digenic and oligogenic inheritance modes were first reported by Dodé et al. in a KS patient with mutations in both *PROKR2* (p.L173R) and *ANOS1* (p.S396L) [50]. Application of NGS has promoted the discovery of digenic and oligogenic inherited genes in KS patients, which also increases the complexity for genetic counseling.

### Differential Diagnosis of KS

Despite the diagnostic criteria mentioned above, the key point for KS diagnosis is to clarify the differential diagnoses. The main differential diagnoses of KS include tumors caused by acquired hypogonadotropic hypogonadism, constitutional delay of growth and puberty (CDGP), CHARGE syndrome, and functional hypogonadotropic hypogonadism [28, 60, 61]. Among these differential diagnoses, CDGP is the most challenging, because KS and CDGP are both associated with delayed puberty. Delayed puberty is defined as delayed puberty onset or progression by >2–2.5SD compared with the population mean, including absence of testicular enlargement (<4 mL) at 14 years of age in boys and absent breast development at 13 years of age in girls [62]. Differences in testicular size, presence of cryptorchidism and/or micropenis, CHH-associated phenotypes, and genetic testing can provide partial diagnostic evidence to distinguish between KS and CDGP [28].

Normosmic congenital hypogonadotropic hypogonadism (nCHH) shares similar phenotypes with KS, including bimanual synkinesia, renal anomalies, facial cleft, dental agenesis, eye movement disorder, and hearing loss, although the prevalences of these abnormalities are more common in KS than in nCHH [63, 64]. Thus, it is difficult to distinguish between nCHH and KS based on clinical phenotypes alone. Genetic analyses have a crucial impact on diagnosis for the two diseases. Some overlapping genes have been identified between nCHH and KS, including *FGFR1*, *FGF8*, *FGF17*, *NSMF*, *SPRY4*, *IL17RD*, *DUSP6*, *GLCE*, *FLRT3*, *PROK2/PROKR2*, *HS6ST1*, *HESX1*, *CHD7*, *KLB*, *PLXNA1*, *WDR11*, *DCC*, *NTN1*, *SEMA7A*, and *SEMA3E* [63]. Besides these overlapping genes, *OTUD4*, *RNF216*, *POLR3A*, *POLR3B*, *PNPLA6*, *PITX2*, *STUB1*, *SOX2*, *SOX3*, *OTX2*, *PROP1*, *PCSK1*, *LHB*, *FSHB*, *LHX4*, *LHX3*, *DMXL2*, *GNRH1*, *GNRHR*, *GLI2*, *GATA2*, *IGSF10*, *KISS1*/*KISSR1*, *TAC3*/*TACR3*, *LEP*/*LEPR*, *TBX3*, *NR0B1*, and *CCDC141* gene mutations were only reported in nCHH [63]. The combination of clinical symptoms and genetic testing could improve the accuracy of diagnosis to some extent, especially for the patients without overlapping genes. However, overlapping gene mutations may require further explorations for the patients with confusing clinical symptoms. The different clinical manifestations may be related to the variation types of the genes, variations at different gene functional domains, the digenic and oligogenic modes, and even the environmental influence.

Adult-onset hypogonadotropic hypogonadism (AHH) occurs in healthy adult males who have completed normal puberty and have proven fertility [65]. AHH can be caused by anatomic etiologies, infiltrative diseases, space-occupying lesions, other central nervous system tumors, and genetic disorders [66, 67]. The rare DNA variants identified in AHH patients include mutations in *GNRHR*, *FGF8*, and *PROKR2* [66], which are also found in KS patients.

CHARGE syndrome is a multisystem disorder that includes symptoms of Coloboma, Heart defect, Atresia of choanae, Retardation of growth and/or development, Genital hypoplasia, and Ear anomalies [68]. CHARGE syndrome tends to be sporadic with an incidence of around 1/10,000, but can sometimes show an AD inheritance mode [68]. Mutations in *CHD7* are identified in >75% of patients with CHARGE syndrome. KS and nIHH were hypothesized to be milder allelic variants of CHARGE syndrome [69]. KS and CHARGE syndrome share some similar symptoms and common genetic mutations, such as olfactory abnormalities, hearing loss, genital hypoplasia, cleft lip and palate, hand anomalies, and renal anomalies [68, 70]. Therefore, it is important to distinguish KS and CHARGE syndrome using both clinical symptoms and genetic testing.

### Treatments for KS

Early diagnosis and treatment of KS are crucial, because they can lead to improved quality of life and prevention of disease-related complications, especially during adolescence as the vital period for secondary sexual maturation and functioning and the critical point for adolescent psychological development. As mentioned before, CHH and hyposmia/anosmia are the two main symptoms of KS, especially CHH causes the most disturbed symptom in KS patients’ life. Surgical operative is the first choice for newborns with cryptorchidism at 6–12 months. HRT promotes virilization, development of muscle mass and strength, pubertal growth spurt, deepening of voice, growth of penis, libido, and sexual function in males [20] and furthers development of breast in females. HRT could also improve skeletal maturation and bone mass density, self-confidence, and well-being in both genders. It should be noted that HRT prescriptions are different depending on the main goal and age of KS patients. Low dose of testosterone is used to induce penis growth of male newborns after surgery. For adolescent males lacking puberty, sex steroid therapy (testosterone in KS male patients) or gonadotropin (including human chorionic gonadotropin (hCG) and follicle-stimulating hormone (FSH)) is used for pubertal induction and development of normal secondary sexual characteristics [20]. To recover the fertility in KS adult males, pulsatile GnRH and gonadotropin are used for treatment. In adolescent girls, 17β-estradiol is administrated to induce puberty, and progesterone is added during the last 14 days of menstrual cycle after breakthrough bleed or full breast development [28, 71]. Estrogen-progesterone therapy is also recommended for the KS females without fertility intention [71]. For the women with fertility desire, pulsatile GnRH/gonadotropin treatment is suggested to mimic physiological condition [71, 72]. After timely and appropriate HRT, KS patients can develop secondary sexual characteristics, maintain normal sex hormone levels, lead a healthy sexual life, and achieve fertility. KS patients generally require lifelong treatment; surprisingly, however, 10–20% patients show spontaneous reversal of reproductive function, although subsequent relapse can occur [73, 74].

Although current treatments markedly improve quality of life in KS patients, the genetic characteristics of KS mean that there is some risk of transmitting the mutated genes to offspring and presenting severe symptoms.

## Advances in Genetic Interruption for KS in Clinical Settings

### Prenatal Diagnosis of KS

Prenatal screening/diagnosis is an effective method in clinical settings to decrease birth defects and block transmission of genetic disorders. Different methods can be used, including maternal serological screening markers, fetal chromosome karyotype analysis, fetal ultrasound, and non-invasive prenatal testing [75]. KS fetuses can be identified through prenatal diagnosis, such as the case of a male fetus with X-linked recessive chondrodysplasia punctata, steroid sulphatase deficiency, X-linked KS, and chromosome deletion at Xp22.31 [76]. Ultrasound showed nasal hypoplasia with a depressed nasal bridge, short septum, and deep groove between the tip of the nose and the alae nasi, before termination of pregnancy at 19 weeks. Autopsy further showed the absence of bilateral olfactory bulbs and tracts in the brain. Although prenatal diagnosis during pregnancy can help to decrease birth defects, pregnant women suffer from physical and psychological distress when fetuses are diagnosed with congenital defects.

### Perspectives for PGT Application in KS Patients

Although the majority of infertile male and female patients with KS have a good reproductive prognosis after appropriate HRT or through assisted reproductive technologies (ART), all KS patients who seek fertility should receive counseling and discuss with their doctors or genetic specialists about the risk of transmitting the disease to their offspring [20, 58, 77]. Considering the genetic characteristics, risks of transmission to their offspring, and possibility of causing defects, KS patients and their families are recommended to evaluate these risks and receive genetic counseling.

In recent years, besides Sanger sequencing and other traditional methods used for PGT, the booming development of high-throughput NGS technology had led to its common use in PGT, providing improved resolution and increased genetic information and reducing the cost and time required for sequencing [78]. Multiple Annealing and Looping-Based Amplification Cycles (MALBAC) is a new whole-genome amplification method which uses quasi-linear preamplification to reduce the bias associated with nonlinear amplification and can achieve 93% genome coverage at ≥1× for a single human cell at 25× mean sequencing [79, 80]. Besides improvements in the MALBAC technology, its combination with linkage analyses has provided a cost-effective method in clinical settings, called Mutated Allele Revealed by Sequencing with Aneuploidy and Linkage Analyses (MARSALA) [81]. MARSALA can achieve simultaneous detection of variant sites and chromosomal abnormalities and linkage analysis of embryos in a single procedure and reduce the amplification bias [80–82]. Owing to its advantages of multiple-level genome proofreading, high accuracy, comprehensive analysis, low cost, and easy operation, MARSALA-PGT is commonly used in clinical settings [81]. To date, MARSALA has been successfully used in PGT for AR, AD, and X-linked recessive disorders, including beta-thalassemia [82], spinal muscular atrophy [83], polycystic kidney disease [84], primary open angle glaucoma [85], Marfan syndrome [86], and retinitis pigmentosa [87].

In 2019, Chan et al. reported the first live birth of a term baby girl delivered into a family wherein the husband had KS with anosmia and absence of secondary sexual characteristics [88]. An FGFR1 heterozygous variation (valine to isoleucine) was found in the male patient and his father, but his father was not affected by KS, and the variation was considered most likely to represent a polymorphism. This male KS patient received treatment with human chorionic gonadotropin and human menopausal gonadotropin, ICSI (due to low sperm count), and PGT for aneuploidy (PGT-A) [88]. After two cycles of euploid embryo transfer, his wife eventually achieved pregnancy. Because of the uncertainty regarding the pathogenic gene, PGT for monogenic disease (PGT-M) was not performed.

In this reported case, only 30 KS- or CHH-related genes were tested, and the *FGFR1* mutation was found in both the patient and his father. However, the father did not show any symptoms of KS, which may be related to incomplete penetrance and variable expressivity among different individuals or oligogenicity. For this family, whole-exon sequencing would be required to identify other potential pathogenic genes. Thus far, genetic causes can be determined in 50–60% of all KS cases, which is vital for diagnosis and fertility prognosis of KS patients, and PGT has become an optional approach to block transmission among generations.

With the current improvements in its efficiency and security, PGT has become a useful approach for familial inherited diseases to prevent transmission of pathogenic genes to the following generation. However, thus far, KS patients have very rarely chosen to acquire their babies through PGT procedures in clinical settings. This may arise because the majority of male KS patients can produce sperm and acquire offspring after a regular period of HRT, the symptoms are mild in some KS patients, or there is economic pressure. The majority of KS patients choose to acquire their offspring by natural fertilization or IVF after HRT. For patients with family histories, genetic testing and counseling are recommended to establish the mutated genes, associations between pathogenic genes and phenotypes, and inheritance patterns. In families with severe inherited symptoms, such as congenital heart defect, hearing loss, and cleft lip/palate, PGT can be an optional approach to avoid offspring suffering from KS. However, in some cases, there are some limitations and challenges for genetic counseling caused by incomplete penetrance, and oligogenicity for known and unknown genes. Therefore, PGT is a perspective genetic interruption method for KS. Both methods have their advantages and disadvantages, so that it is difficult to evaluate which one is obviously better than the other. For PND, fetal samples are obtained by invasive methods (such as chorionic sampling, amniocentesis, and umbilical vein puncture) for genetic prenatal diagnosis to find whether the fetus carries genetic abnormalities. But the pregnant woman may suffer from psychological stress if the fetus has been diagnosed with KS. While, PGT can avoid such a situation by selecting and transfer an embryo without genetic abnormalities, but OHSS may occur during PGT procedures, and the biopsy procedure is invasive and may be harmful to the embryos.

**Fig. 1:**
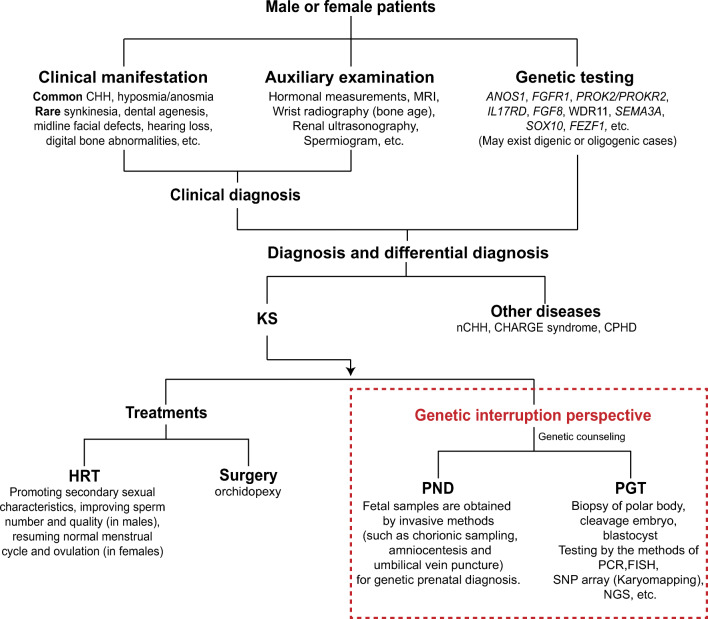
Diagram for clinical management and genetic interruption perspective of KS

The current clinical management and genetic interruption perspective of KS are summarized in Fig. 1.

In conclusion, KS is a rare hereditary disease with high phenotypic and genetic heterogeneity. In the last decade, pathogenic genes have gradually been identified in KS patients. Although appropriate HRT or ART can help infertile male and female patients to achieve a good reproductive prognosis, their offspring still have risks of suffering from KS. Although a prenatal diagnosis can detect congenital defects during pregnancy, pregnant women may suffer from physical and psychological distress, especially when the fetus is found to be carrying genetic defects. Meanwhile, with the advances and application of NGS and single-cell amplification technologies, as well as improvements in safety and accuracy, PGT has helped thousands of families with hereditary diseases to obtain a healthy next generation. Although no successful case with genetic interruption of PGT to KS patients was reported until now, genetic counseling is an essential component for KS patients who seek fertility, and it can be an imaginable outlook that PGT becomes an optional approach in the near future to decrease the risk of transmitting the disease of KS to their offspring.
